# Electrical characterization of DNA supported on nitrocellulose membranes

**DOI:** 10.1038/srep29089

**Published:** 2016-07-12

**Authors:** Mahmoud Al Ahmad, Reham M. Milhem, Neena G. Panicker, Tahir A. Rizvi, Farah Mustafa

**Affiliations:** 1 Department of Electrical Engineering, College of Engineering, United Arab Emirates University, Al Ain, UAE; 2 Zayed Bin Sultan Center for Health Sciences Division, United Arab Emirates University, Al Ain, UAE.; 3 Department of Biochemistry, College of Medicine and Health Sciences, United Arab Emirates University, Al Ain, UAE.; 4 Department of Microbiology and Immunology, College of Medicine and Health Sciences, United Arab Emirates University, Al Ain, UAE.

## Abstract

Integrated DNA-based nanoscale electronic devices will enable the continued realization of Moore’s Law at the level of functional devices and systems. In this work, the electrical characterization of single and complementary base paired DNA has been directly measured and investigated via the use of nitrocellulose membranes. A radio frequency DAKS-3.5 was used to measure the reflection coefficients of different DNA solutions dotted onto nitrocellulose membranes. Each DNA solution was exposed to a radio frequency signal with a power of 10 dBm and with a sweep from 200 MHz up to 13.6 GHz. The conducted measurements show some distinctions between the homomeric and complementary bases due to their different electrical polarization. As revealed from the measurements conducted, with the addition of DNA oligonucleotides, the measured capacitance increased when compared with buffer medium alone. The DNA molecules could be modeled as dielectric material that can hold electrical charges. Furthermore, the complementary paired DNA molecule-based inks solutions had a higher capacitance value compared with single DNA molecules (A, C, G and T) solutions.

The use of DNA in hybrid biosensors which combine electrical parameters and biological properties target a desired functionality in smart microsystem technologies^1^,^2^. Their use in biomedical and environmental applications is attracting great interest^3^. Moreover, the recent advancements in detection, quantification and identification of biotic materials are keys for enabling lab-on-chip integrated DNA thin film layer^4^ and ink-based inject systems^5^. Organic electronic devices use thin films of DNA in their applications to allow light-emitting diodes to shine brighter and last longer^6^. These ultra-thin DNA organic films have found their way into sensors and optic design^7^. DNA thin films on polystyrene microspheres have proven useful for the delivery of DNA-encoded antigens to macrophage cells and provide new materials-based methods for the design and delivery of DNA vaccines^8^. The fabrication of multi-layered DNA platforms allow the controlled release or even localized delivery of DNA or other nucleic acid-based materials^9^.

Paper-based diagnostics such as porous nitrocellulose membranes have been used for the direct absorption of biomolecules such as immunoglobulin G (IgG) antibodies on their surface for analyte detection^10^,^11^,^12^,^13^. As a porous material, it is able to immobilize substrates such as proteins and/or nucleic acids by direct physical absorption. Immobilization approaches have been demonstrated on microarrays^14^ and cellulose-based assays^15^ and microfluidic devices^16^. These approaches, however, have not been carried out on nitrocellulose membranes^10^. Further applications– the substrate’s ability to covalently attach proteins and direct coupling with an epitope or probe^10^ has led to its use in bioassays such as dot blots and lateral flow immunoassay tests. Its use as a low-cost, disposable material has found its way in the detection of breast cancer cells^17^, and in the screening of drugs^18^. Due to its small pore size (0.1–0.45 μm) and high binding capacity (80–100 μg/cm^2^), along with its high frequency performance, all these attributes make it a good substrate to be used for the characterization of DNA molecules.

Taking into account the potential use and unique immobilization property of nitrocellulose, this work was undertaken to merge the research on paper, which can be used as a dielectric substrate, with several DNA-based inks to measure and characterize the electrical performance of DNA. Thus, the current research was directed towards low-cost, disposable, and portable paper-based diagnostics, with the aim of addressing the performance of nitrocellulose paper as a substrate in the electrical analysis of DNA oligonucleotide fragments. DNA on nitrocellulose paper is a general approach that could, with further development, aid in the preparation of thin films for several electronic, electro-optic and novel delivery applications.

## Theoretical Background

**Figure 1:**
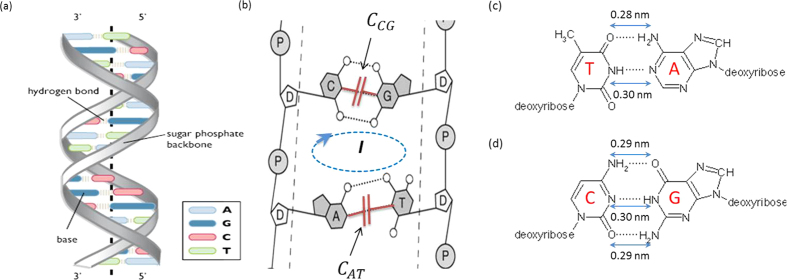
DNA double strand structure and modeling. (**a**) Anti-parallel double strand DNA showing the base pairing of AT and CG. (**b**) Electrical modeling incorporating the electrical capacitances and current flow. (**c**) Pairs of complementary nucleosides present in double-stranded AT DNA molecules along with geometrical dimensions of their hydrogen bonds shown as “….”. (**d**) Pairs of complementary nucleosides present in double-stranded CG DNA molecules along with geometrical dimensions of their hydrogen bonds shown as “….”.

DNA is a double stranded molecule^19^ shown in Fig. 1(a) composed of two anti-parallel chains that consist of a sugar (deoxyribose, D) and phosphate (P) backbone. The sugar molecule has five carbon atoms of which carbon number 3 and 5 are involved in covalent bond formation with the phosphate group. The direction of the two chains (one in the 5′ to 3′ direction and the other in the 3′ to 5′ direction) is determined by the carbon number within the sugar molecule. The two sugar-phosphate backbones of DNA are linked to each other via the nitrogenous bases that form hydrogen bonds between complementary sets of bases: thymine (T) base pairs via two hydrogen bonds with adenine (A), while cytosine base pairs with guanine (G) via three hydrogen bonds.

The electrical representation of capacitance and current flow within a DNA molecule are depicted in Fig. 1(b). It is these hydrogen bonds (mutually-bonding electrical forces) that complete the electrical circuit in a DNA molecule^20^. Thus, the electrical model of a DNA molecule can be visualized as containing two different types of capacitors between the complementary nitrogenous bases (*C*_*AT*_ and *C*_*CG*_ shown in red) that create electrical circuits between the two strands of DNA. The clockwise rotational arrow points to the helical nature (rotational symmetry) of the two twisted anti-parallel strands of the double-stranded DNA molecule as well as the current flow (*I*) within the electric circuit^20^.

In principle, a double-stranded DNA molecule can be considered as a dielectric material that can be polarized with the application of an electric field, enabling investigation of its electrical properties^21^. As shown in Fig 1(c,d), the O and N atoms hold negative charges, while the H atom holds a positive charge. Furthermore, within the double-stranded DNA, there are two types of chemical bonds: the O-H bond (O from the thymine molecule and H from the adenine molecule) and the NH-N bond (NH from the thymine molecule, N from the adenine). It is estimated that the distance between the atoms on each molecule is 0.1 nm^22^. Thus, the two strands of the double-stranded DNA are held together by electrostatic forces due to the net average charge between the H and N atoms and the C and O atoms, which is calculated to be 0.2e and 0.4e, respectively. The distance between N-H-N bonds is approximately equal and estimated to be 0.3 nm, whereas the O-H-N bonds in AT and GC are 0.28 nm and 0.29 nm, respectively^23^.

Thus, when a voltage is applied to a solution of DNA, an electrical current should propagate between the applied voltage terminals, resulting in a local current flow inside the DNA molecule, causing the capacitances to get charged^24^. In a DNA suspension, depending upon the nature of the DNA molecules, multiple single/double-stranded DNA molecules are randomly distributed. An applied electric field polarizes these strands. The intrinsic properties of the DNA strands determine the strength of the polarization which measures its ability to hold electrical charges. When the DNA molecules are charged, it behaves as an electrical semiconducting dipole which can induce current in the DNA loops, as suggested.

## Experimental Design

To test the electrical properties of DNA molecules supported onto the solid nitrocellulose membrane, four small molecules of DNA of known size and chemical composition were synthesized commercially. Each of the DNA molecule consisted of 18 building blocks of DNA, deoxyribonucleotides, consisting of either deoxyadenosine (dA), deoxythymine (dT), deoxycytosine (dC), or deoxyguanine (dG) phosphates. The four types of 18-mer individual molecules of DNA oligonucleotides were suspended in a buffer composed of 10 mM Tris, 1 mM EDTA, pH 8 buffer (TE) at a concentration of 4 μM and used to spot the nitrocellulose membrane. The DNA oligonucleotides were spotted either individually, or the suspensions were used to artificially create double-stranded DNA molecules using the ability of the complementary bases to “anneal” and form hydrogen bonds between the two strands of oligonucleotides, as described earlier.

**Figure 2:**
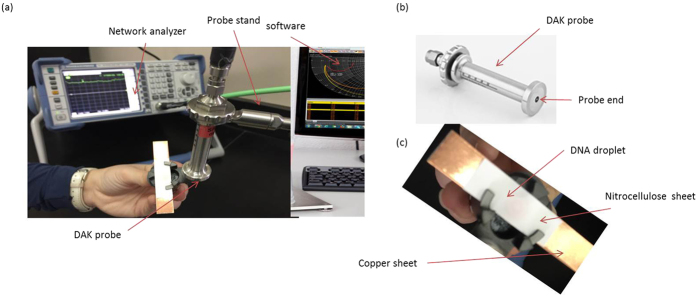
Depiction of the DAKS measurement setup. (**a**) The entire set up consists of the network analyzer, the probe, along with its stand holding the nitrocellulose membrane. A close-up view of (**b**) the DAK probe and (**c**) the stand holding a copper sheet supporting the nitrocellulose membrane spotted with a solution of DNA.

The experimental setup to test the electrical properties of the single and double-stranded DNA molecules is depicted in Fig. 2(a). It used the dielectric assessment kit (DAKS-3.5) probe^25^ to measure the reflection coefficient of DNA molecules spotted onto a nitrocellulose membrane. The DAKS measurement system is a user-friendly kit to assess the dielectric properties of biological, chemical, and electrical materials using a combination of the DAK-3.5 probe and a reflectometer. The reflectometer measures the reflection and transmission of electrical networks at high frequencies. The DAKS probe is connected to a network analyzer (R&S ZVL)^26^ and the system is controlled by the DAK software. Each DNA suspension is exposed to a radio-frequency signal with a power of 10 dBm and with a sweep from 200 MHz up to 13.6 GHz (the measurement capability of the equipment). The reflectometer communicates directly with the DAKS software via a USB port. The DAK-3.5 is the precision dielectric probe used for measurements over the 200 MHz–20 GHz frequency range Fig. 2(b). This open-ended coaxial probe uses advanced algorithms and hardware to measure the dielectric properties of suspensions over a broad range of parameters. The measurement method is fast and non-destructive to the material being tested. Calibration is performed according to SPEAG’s standards^25^. The system is calibrated using the short-load-open reflection line techniques for the network analyzer^27^ to ensure that whatever is measured is representative only of the input solution. A typical calibration moves the measurement reference planes to the end of the probe. Therefore, it excludes the effects of losses and phase shifts that could add noise to the measured signal. The DNA suspensions were spotted onto the nitrocellulose membrane using the same volume of 20 μl that formed a disc of ~5 mM diameter, as shown in Fig. 2(c). To conduct the RF measurements, the nitrocellulose sheet was supported using a copper -block attached to the DAK probe.

## Results and Discussion

**Figure 3:**
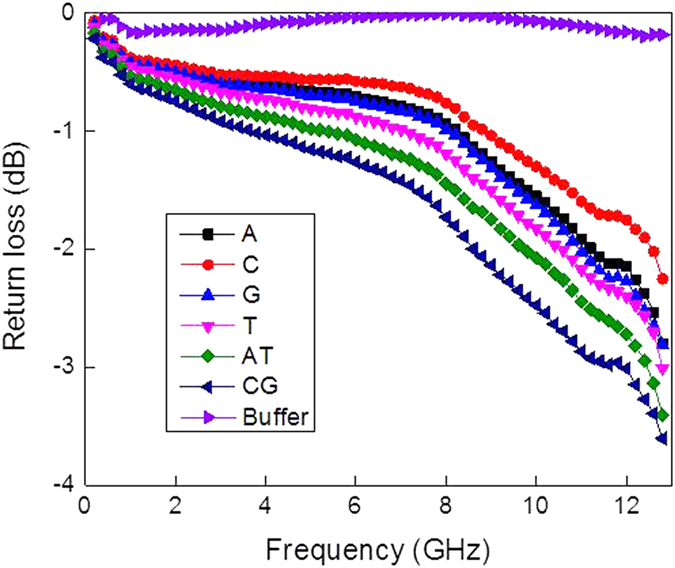
DAK electrical measurements (reflection coefficients versus frequency) of the single- and double-stranded DNA suspensions spotted onto nitrocellulose membranes compared to the control buffer lacking any DNA.

Electrical characterization of the single- and double-stranded DNA oligonucleotides immobilized onto the nitrocellulose surface was conducted as described. Figure 3 reveals the reflection coefficients versus frequency of the DNA oligomers along with the control annealing buffer spotted on the nitrocellulose membrane. When compared with the buffer alone, the presences of DNA molecules increased the reflection coefficient magnitude, revealing that the resistance of the DNA solution decreased with the presence of DNA. The measured reflection coefficients showed a smooth behaviour over a wide frequency range.

The corresponding effective capacitances (*C*) of the DNA suspensions were extracted from the measured reflection coefficients using the relationships defined below^28^:


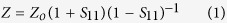



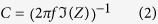


where *Z*, *Z*_*o*_, *S*_11_ are the input impedance, the characteristic impedance of 50 ohms and the reflection coefficient, respectively. *f* is the frequency and 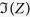 is the imaginary part of the input impedance. At the frequency range between 200 MHz up to 13.6 GHz, the input impedance imaginary part is negative and exhibits a strong capacitive behaviour, while the measured effective inductor is very small (~0.01 μH) which can be neglected.

**Figure 4:**
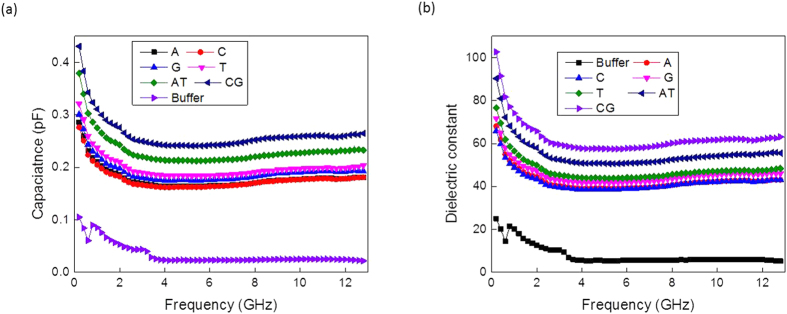
Capacitances and dielectric measurements: (**a**) capacitance versus frequency and (**b**) corresponding dielectric constant versus frequency.

The extracted capacitances of the different DNA suspensions versus frequency are plotted in Fig. 4(a). The capacitance is inversely proportional to the frequency; as the frequency increases the capacitance value decreases. The measured capacitances show a smooth behaviour versus frequency. As revealed from Fig. 4(a), the control buffer showed the lowest capacitance value, while with the addition of DNA oligomers, the measured capacitance increased. The DNA molecules could be modeled as dielectric material that can hold electrical charges. Figure 4(a) further showed that the double-stranded DNA molecules had a higher capacitance value compared to the single-stranded DNA molecules (A, C, G and T). Moreover the CG base pair showed a higher capacitance value compared to the AT base paired DNA. The magnitude of electrostatic forces between the complementary pairs were calculated using Coulomb’s law^29^, which revealed that the force between the GC pair is 69.12 nN, and between the AT pair is 46.12 nN. Therefore the higher the force indicates higher amount of charges and implies higher capacitance. Thus, our findings showing that the capacitance of CG is higher than that of the AT base pair is consistent with these facts.

Next, the corresponding dielectric constants for the suspensions were computed form their capacitance values using the following equation^28^:


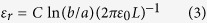


where *b*, *a* and *L* are dimensions of the DAK probe-the outer, inner radius and the length, respectively. *ε*_0_ is the dielectric constant of the space. The extracted dielectric constants versus frequency were plotted and are shown in Fig. 4(b). The frequency response of the dielectric constant is conformally mapped with the corresponding capacitances.

The contribution of the control buffer was next eliminated by de-embedding it from the capacitance values of the DNA suspensions. This de-embedding step was conducted by subtracting the capacitance value of the control buffer from those of the DNA samples (DNA printed solutions) due to that fact that buffer contribution could act as a linear superposition to that of the DNA samples. Equivalently, the capacitance value of the control was considered in parallel to that of the sample. This “parallel model” was based on the observation that the effective capacitance of the DNA suspension (DNA + buffer) was higher than the capacitance of the DNA-free control medium. If the effective capacitance of the printed solution was lower than the reference, then a “series model” would have been considered.

**Figure 5:**
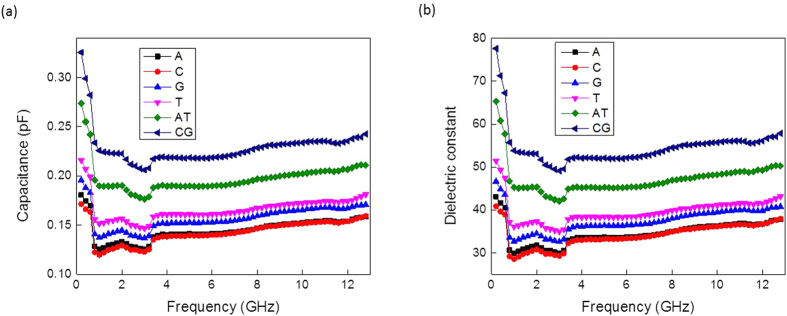
Results of electrical characterization of DNA after de-embedding the effects of the control buffer: (**a**) capacitance and (**b**) dielectric constant versus frequency.

Figure 5(a) depicts the capacitance values of single- and double-stranded DNA molecules versus frequency after de-embedding the contributions of the control buffer. As can be seen clearly, it was easier to differentiate the double-stranded DNA molecules from the single stranded ones after the de-embedding step since the single stranded DNA exhibited its own capacitance profile even after subtracting the effects of the buffer medium. The corresponding dielectric constants after the de-embedding step are shown in Fig. 5(b). The four single DNA oligomers showed lower dielectric values compared to their base-paired counterparts. Furthermore, these profiles exhibited a step-like decrease between 1 and 3 GHz which is probably due to the buffer frequency response. We believe that with more frequency measurements, the capacitance profiles exhibited should become continuous. (The maximum numbers of frequency points measured were determined by the network analyzer).

The dielectric constant of DNA is not well known because of the obvious difficulty of its direct measurement. Different values for dielectric constant are proposed in different works, showing a discrepancy and inconsistency and has been the subject of conjecture. A low value for DNA dielectric constant of 2 was suggested in^30^, other larger values of 12.4^31^ and the highest ever reported was 100^29^. Mazur *et al*. have assumed a low value for the DNA dielectric constant of 2 in their analysis of the electrostatic contributions to DNA base-stacking interactions^30^. Tavernier *et al*. have suggested that the dielectric constant of pyridine is 12.4 which provide a more realistic approximation, as per their opinion^31^. Williams *et al*. have reported an estimated value of 100, which was extracted using a one-dimensional point charge model^32^.

**Figure 6:**
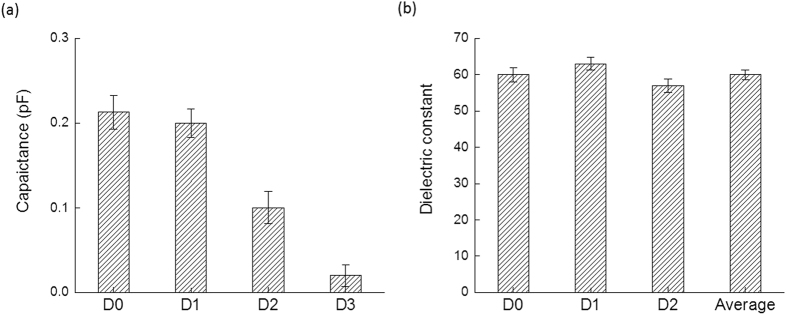
Sensitivity testing of DNA solution supported on nitrocellulose membranes. The double-stranded AT DNA solution was serially-diluted 10-folds and subjected to blotting onto a nitrocellulose membrane followed by its electrical characterization using the DAK set up. (**a**) Capacitance and (**b**) dielectric constants of the serially-diluted AT base paired DNA at 5 GHz.

Finally, the sensitivity of the electrical method was tested by diluting known concentrations of double-stranded AT base paired solution ten-folds serially with its relative control buffer. As shown in Fig. 6(a), the more concentrated the DNA solution, the higher the observed capacitance values. This was followed by extraction of the dielectric constants of the serially-diluted AT solutions. As expected, since dielectric constant is an intrinsic property of the material, the extracted dielectric constants of the diluted AT DNA were observed to be close to each other after the deembedding step of the buffer despite the ten-fold dilution. The average value of the dielectric constants observed with each dilution along with their standard deviation is depicted in Fig. 6(b).

In further agreement with our findings, previously, DNA impedance measurements studies have been conducted to examine the impact of length and concentration of free-floating double-stranded DNA molecules^24^. The suspension parameters were extracted by fitting the impedance versus frequency characteristics to an equivalent circuit model. An AC voltage with amplitude of 250 mV was applied to the electrodes and impedance was measured with frequency varying from 100 Hz to 1 MHz. Based on extraction of circuit model parameters, it was demonstrated that the changes due to concentration and sizes of DNA are directly related to the total number of base pairs in the solution and the conductance and the capacitance of the solution increased with DNA concentration and length.

### Biochemical analysis of DNA Oligonucleotides

**Figure 7:**
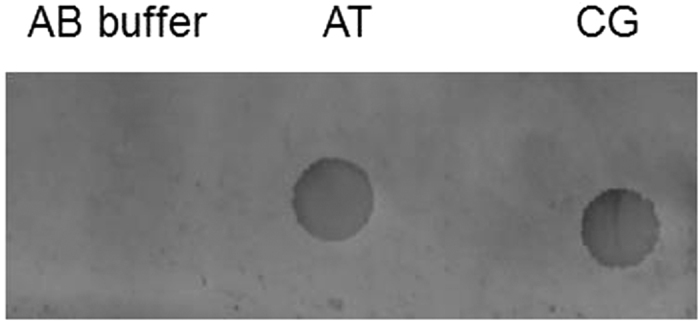
Dot-blot of the oligonucleotides stained with SYBR^®^ Gold stain following UV crosslinking onto the nitrocellulose membrane. AB buffer = Annealing Buffer; AT and CG are the two types of double-stranded DNA molecules tested in this study.

To validate the presence of the oligonucleotides biochemically on the nitrocellulose membrane after performing the electrical parameters, the DNA solutions were immobilized onto nitrocellulose membrane by UV crosslinking and then visualized by staining with the sensitive SYBR^®^ Gold stain (Fig. 7). Once bound to DNA, SYBR^®^ Gold stain^33^ increases its fluorescence over a thousand fold that can be detected by Typhoon FLA 9500. As can be seen, the double-stranded oligonucleotides could be visualized as dots on the nitrocellulose membrane; however, the single-stranded oligonucleotides could not be visualized, most likely due to the small size of the molecules (only 18 nucleotides long), preventing their staining with the dye.

**Figure 8:**
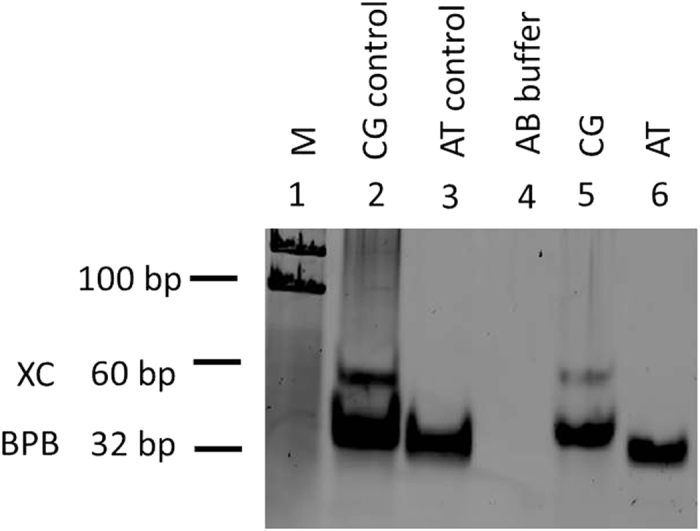
Image of the oligonucleotides stained with SYBR^®^ Gold nucleic acid stain following elution from nitrocellulose membrane and size fractionated using a 15%polyacrylamide gel. A 100 bp molecular weight ladder (M) shown in lane 1 was used to monitor the size of the oligonucleotides alongside two other internal dyes, xylene cyanol (XC) which ran at around 60 bp and the bromophenol blue (BPB) which ran at around 32 bp. The two controls CG and AT analyzed in lanes 2 and 3, respectively, represent DNA suspensions of equal volume that were directly loaded on the gel without having been eluted from the nitrocellulose membranes. AB buffer in lane 4 served as a negative control. Lanes 5 and 6 show CG and AT DNA eluted directly from the nitrocellulose membranes. The amount of CG and AT eluted from the membrane was estimated to be 61% and 64%, respectively.

Another independent technique was used to confirm the presence of DNA in the solutions. The DNA oligonucleotides tested in the electrical experiments were directly eluted from the membrane as described in the experimental procedures. The eluted DNA was precipitated from the elution buffer using the classical ethanol precipitation, re-suspended in AB buffer, diluted in 6X loading dye and loaded on a 15% polyacrylamide gel. Following electrophoresis, the gel was stained with SYBR^®^ Gold stain and the eluted and size-fractionated DNA visualized by UV transillumination (Fig. 8). The size of the oligonucleotides was monitored using a 100 bp molecular weight ladder (M) loaded alongside the samples. As expected, the CG and AT double-stranded DNA molecules could be detected as discrete bands with the CG double-stranded DNA showing a slightly higher molecular weight than the AT band. AB buffer that lacked DNA served as a negative control. An additional minor band of higher molecular weight size could be observed in the CG lane, most likely representing a higher molecular weight DNA oligomer. Once again, the single stranded oligonucleotides could not be observed due likely to the small size of the DNA molecules, but the presence of the double-stranded DNA molecules confirms that DNA was present in the solution.

The amount of CG and AT nucleic acid eluted from the nitrocellulose membranes was estimated using Image J software^34^ and was normalized against the negative control (AB buffer). Compared to the control CG and AT, the amount of CG and AT eluted from the membrane was estimated to be 61% and 64%, respectively.

## Conclusion

In summary, this study reports investigation of detecting unique DNA molecules immobilized onto nitrocellulose membranes. In principle, DNA nucleotides can be considered as complex particles that can be polarized with the propagation of high frequency signals. Our results and findings reveal that double-stranded DNA has higher dielectric constant compared to single-stranded DNA. Moreover the CG base pair showed a higher capacitance value compared to AT DNA base pair. Together, these observation are of potential value towards the development of not only novel electronic devices that incorporate DNA as a new materials, but also towards the use of high frequency techniques to identify the four unique DNA sequences despite their close electronic structure and common properties. Our approach provides significant advancement in the technical know-how and contributes to a new way to check the efficiency of DNA immobilization as well as to generate interest in both experimentalists (device applications) and theorists towards modelling of charge transport in DNA-based devices.

## Materials and Methods

### Reagents

Protran nitrocellulose membrane with 0.45 μm pore size and 300 mm thickness from (GE Healthcare Life Sciences, UK) was used in this work. Tris-base (T6066, Sigma-Aldrich, USA), EDTA (E-5134, Sigma-Aldrich, USA), Tris/Borate/EDTA (10XTBE) buffer (V4251, Promega, USA), 6X loading dye (13526, Norgen, Canada), 100 bp ladder (CSL-MDNA-100 bp, Wolf Laboratories, UK), TEMED (161-0801, BioRad, USA), APS (7727-54, Sigma-Aldrich, USA), 30% acrylamide (A-9099, Sigma-Aldrich, USA), ammonium persulfate (A3678, Sigma-Aldrich, USA) and SYBR^®^ Gold nucleic acid dye (ThermoFischer Scientific, UK).

### Oligonucleotide annealing conditions

To observe the electric properties of double-stranded DNA of specific size and composition, four single-stranded oligonucleotides composed of 18 nucleotides (18-mer) of each type of base, A, T, C, and G were obtained commercially (Macrogen Inc., South Korea). The lyophilized oligonucleotides were resuspended in 10 mM Tris, 1 mM EDTA, pH 8 (TE buffer) followed by their dilution in annealing buffer to a working concentration of 100 μM each. This was achieved by mixing equal volumes of single complementary oligonucleotides (A and T/C and G) using an annealing protocol from Metabion, Germany. Briefly, the resuspended complementary oligonucleotides were mixed at equimolar concentrations (4 μM each) in an annealing buffer (10 mM Tris, 1 mM EDTA and 100 mM NaCl, pH 7.5) and heated at 94 °C for 10 minutes to properly denature the single-stranded oligonucleotides. This was followed by slow cooling at room temperature for one hour to allow the spontaneous generation of double-stranded DNA molecules via hydrogen bonding between complementary bases. The annealed oligonucleotides were aliquoted and stored at −20 °C until further analysis.

### Elution of the DNA oligonucleotides

Following the measurements on the DNA oligonucleotides spotted on the nitrocellulose membranes, the dot of DNA was cut out using a clean blade and placed individually in micro centrifuge tubes containing 400 μl of TE buffer at room temperature. DNA was eluted from the nitrocellulose by shaking the tubes in a shaker at room temperature (1400 × *g*) for 30 minutes. The micro centrifuge tubes were spun at 13000 × *g* and the membrane consequently removed. The eluted oligonucleotides were then precipitated via adding two volumes of ice cold ethanol and cooling the suspension at −80 °C for four hours. The DNA/ethanol solutions were then spun at 13000 × *g* for 30 minutes at 0 °C. The supernatant was discarded and the pelleted DNA was washed with 95% ethanol and air dried. The dried pellets were resuspended in 20 μl of AB buffer analyzed via polyacrylamide gel electrophoresis as outlined below.

### Dot Blot of DNA suspensions

DNA oligonucleotides suspensions were spotted on nitrocellulose paper (20 μl per spot) and cross-linked using a UV cross linker (BLX-254 Cross-linked, Life Technologies, UK) for approximately 60 seconds. The cross-linked membranes were blocked with 3% bovine serum albumin (BSA) to prevent further binding of non-specific DNA in 0.05% TBS-Tween for 1 hour. The blocked nitrocellulose membranes were then washed once with 1XTBE and stained using the SYBR^®^ Gold nucleic acid dye diluted in 1XTBE buffer at the recommended dilution. DNA spotted onto stained nitrocellulose filters was visualized by the Typhoon FLA 9500 and ImageQuant TL software (GEHealthcare Sciences, Uppsala, Sweden) using a filter at 473 nm.

### Polyacrylamide gel electrophoresis

The DNA oligonucleotides eluted from the nitrocellulose membranes were analyzed by polyacrylamide gel electrophoresis (PAGE) technique to confirm the presence of double-stranded DNA. Briefly, 15% polyacrylamide gels were prepared in 1XTBE buffer. The annealed DNA samples were diluted with 6X loading dye and then loaded onto the gels. Following electrophoresis, the polyacrylamide gels were stained using the SYBR^®^ Gold dye diluted in 1XTBE buffer at the recommended dilution. PAGE allows separation of DNA according to size, composition, and charge. The double-stranded DNA products were visualized using the SYBR^®^ Gold nucleic acid dye which binds both single- and double-stranded DNA and fluoresces upon exposure to UV light^30^. The gels were stained for 40 minutes in the dark and the DNA was visualized and photographed using the Typhoon FLA 9500 and ImageQuant TL software (GEHealthcare Sciences, Uppsala, Sweden) using a filter at 473 nm.

## Additional Information

**How to cite this article**: Ahmad, M. A. *et al*. Electrical characterization of DNA supported on nitrocellulose membranes. *Sci. Rep.* **6**, 29089; doi: 10.1038/srep29089 (2016).
